# Evaluation of Three Cytomegalovirus IgG Lateral Flow Assays for Rapid Determination of CMV Serostatus

**DOI:** 10.1093/ofid/ofae084

**Published:** 2024-03-13

**Authors:** Laurel Joncas-Schronce, Fatima Ali, Gregory Pepper, Renee D Stapleton, Gordon D Rubenfeld, Michael Boeckh, Ajit P Limaye

**Affiliations:** Vaccine and Infectious Disease Division, Fred Hutchinson Cancer Center, Seattle, Washington, USA; From the Department of Medicine, University of Washington, Seattle, Washington, USA; From the Department of Medicine, University of Washington, Seattle, Washington, USA; Division of Pulmonary and Critical Care Disease, University of Vermont Larner College of Medicine, Burlington, Vermont, USA; Interdepartmental Division of Critical Care Medicine, Sunnybrook Health Sciences Center, Toronto, Ontario, Canada; Vaccine and Infectious Disease Division, Fred Hutchinson Cancer Center, Seattle, Washington, USA; From the Department of Medicine, University of Washington, Seattle, Washington, USA

**Keywords:** cytomegalovirus, serostatus, lateral flow assay, diagnosis

## Abstract

**Background:**

Cytomegalovirus (CMV) serostatus is a major determinant of CMV infection, disease risk, and transplant outcomes. Current clinical serology assays are limited by relatively slow turnaround time, design for batched testing, need for trained personnel, and/or specialized equipment. Rapid diagnostic assays in development have a role in emerging settings, such as critically ill patients, but have not been systematically evaluated.

**Methods:**

We assessed the performance of 3 rapid lateral flow assays (LFAs) for the detection of CMV immunoglobulin (Ig)G antibodies compared with a reference commercially available CMV IgG enzyme-linked immunosorbent assay in residual serum samples from 200 consecutive adults who underwent clinical CMV serology testing. Samples with discrepant results between the LFA and reference assay were tested by a second reference assay. A subset of serum samples was assessed for interoperator variability. Operating characteristics of the QooLabs LFA were separately assessed in plasma samples.

**Results:**

The sensitivity and specificity of the individual LFA assays using serum varied significantly: 86%/83%, 99/93%, and 57/97%, for Healgen, QNow automated reader, and nanoComposix, respectively, compared with the reference assay. Results for the QNow assay were comparable between automated and manual reads. Among a subset of 10 serum samples assessed by 5 individual operators, 44 of 50 (88%) results were concordant. Among 50 plasma samples assessed by the QooLabs LFA, the sensitivity and specificity were 72% and 96%.

**Conclusions:**

The ease of performance, rapid turnaround time, and good operating characteristics provide the rationale for further evaluation of the Qoolabs QNow LFA in specialized settings where rapid assessment of CMV serostatus would be advantageous.

Cytomegalovirus (CMV) is a major pathogen in a broad range of clinical settings of immunosuppression (transplant, HIV, congenital), and CMV serostatus (as determined by the presence/absence of immunoglobulin [Ig]G antibodies to CMV) is an important determinant of risk [1–3]. In addition to immunocompromised host settings, recent studies have demonstrated frequent CMV reactivation and an independent association of reactivation with worse clinical outcomes in seropositive critically ill adults [4, 5]. A recent Phase 2 clinical trial titled “Ganciclovir/Valganciclovir for the Prevention of CMV Reactivation in Acute Injury of the Lung and Respiratory Failure (GRAIL)” (NCT01335932) was conducted to determine whether administration of prophylactic antiviral treatment for CMV reduces plasma interleukin 6 (IL-6) level in immunocompetent adults with severe sepsis or trauma-associated respiratory failure. In that trial, the most common screen failure reason was a delay in the CMV serology result to enroll patients within the required window. Clinical trials of prophylactic antiviral therapy in intensive care unit (ICU) patients require the rapid identification of CMV-seropositive status to facilitate early enrollment after admission, before development of CMV reactivation, which occurs early during the course of critical illness [5]. Thus, rapid and accurate assays to determine CMV serostatus might be helpful in future ICU CMV studies for timely recruitment and enrollment to initiate preventive therapy before CMV reactivation. Current clinical assays for assessing CMV serostatus have several limitations that make them poorly suited for rapid on demand single-sample testing. Some of these limitations include relatively long turnaround time (typically several hours), assay format optimized for batch testing, requirement for trained personnel, and/or requirements for specialized equipment (optical density readers, calibrated pipettes). Large reference laboratories have the capacity for rapid turnaround time (within hours); however, these laboratories are typically decentralized and incur delays in specimen packing and shipment.

QooLabs Inc. and nanoComposix received support from the National Institutes of Health through Small Business Innovation Research funding to develop rapid and easy-to-perform lateral flow CMV serologic assays, which could be well suited to rapidly assess CMV serostatus for various indications, including eligibility for ICU clinical trials as described above. Another rapid LFA kit (Healgen CMV IgG antibody assay) is commercially available but not approved for use in the United States. The sensitivity, specificity, and predictive values of these rapid assays have not previously been systematically assessed. The primary objective of this study was to evaluate the sensitivity and specificity of 3 lateral flow assays (LFAs) that are currently in development in determining CMV serostatus (IgG antibodies) in adults. Secondary objectives were to compare plasma and serum specimens and to assess interoperator agreement.

## METHODS

### Patients/Samples

Residual serum samples (n = 200) and plasma samples (n = 50) from consecutive adults who underwent clinical CMV serology testing at the University of Washington (UW) virology lab were used for this study. Characteristics of the patients included 59.5% male and mean age 48 years. Samples were stored at −80°C until analysis. Aliquots of these samples were tested by a single research lab technician in a blinded fashion (ie, without knowledge of the original clinical result) using the 3 separate CMV lateral flow assays (LFAs), Healgen CMV IgG Rapid Test, Qoolabs’ QNow Rapid Tests for Human CMV Antibody, and nanoComposix IgG LFA, according to the instructions provided by the manufacturer. The QooLabs QNow LFA is commercially available as a “Research Use Only” assay in the United States, while the NanoComposix and Healgen assays are not listed as commercially available on their respective websites.

**Figure 1.**
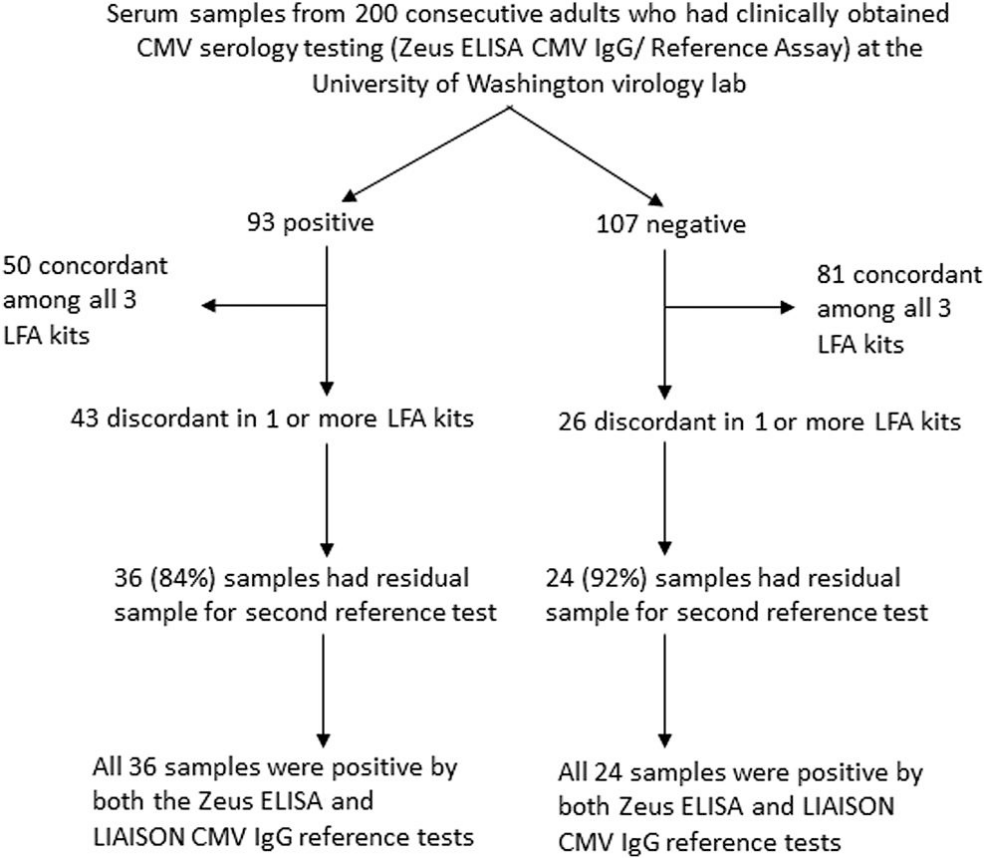
Flowchart of serum sample results breakdown. Serum samples from 200 consecutive adults who had clinically obtained CMV serology testing (Zeus ELISA CMV IgG/Reference Assay) at the University of Washington virology lab. Abbreviation: CMV, cytomegalovirus.

Serum and plasma results were compared with the Zeus ELISA CMV IgG assay as the primary reference assay. A second reference CMV IgG antibody assay (LIAISON CMV IgG) was performed on the subset of serum samples (n = 60) for which there was discordance between the primary Zeus ELISA reference assay and 1 or more of the LFAs (Figure 1). The study was assessed for the need for formal institutional review board (IRB) review and was deemed IRB exempt.

### Reference Assays

The results of each LFA were compared with the primary reference Zeus ELISA CMV IgG assay (manufactured by Zeus Scientific Inc, Branchburg, NJ, USA) and interpreted per the product insert (Zeus ELISA CMV IgG) [6]. The Zeus ELISA test is intended to be used to evaluate serologic evidence of previous or primary infection with CMV, and its index value/OD ratio is interpreted as follows: 0.90 or less (negative), 0.91 to 1.09 (equivocal), 1.10 or greater (positive). Among samples for which there was complete agreement between the 3 LFAs and the primary reference assay, the reference assay result was considered the final result, and additional testing was not performed. For samples that were discordant in any of the test LFAs compared with the primary reference Zeus assay, a second reference assay, LIAISON CMV IgG (manufactured by DiaSorin Inc., Saluggia, Italy), was performed at ARUP Laboratories [7].

### Lateral Flow Assays

**Figure 2.**
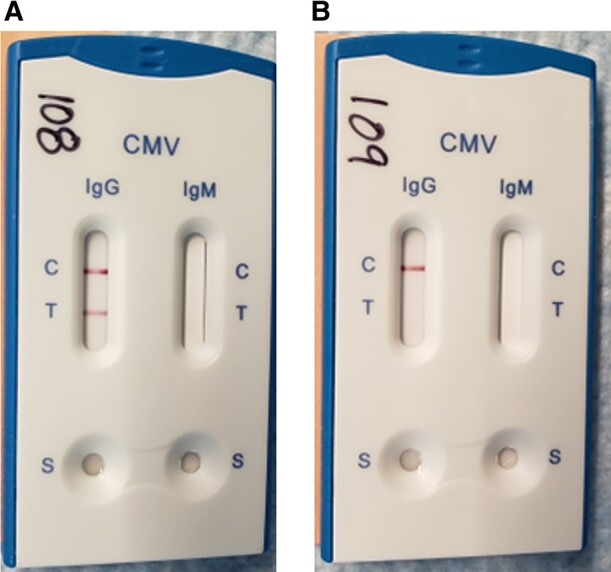
Healgen LFA test strips. Healgen assay results: A, Left cassette indicates presence of CMV IgG in serum by presence of a colored band at both the control (C) region and test (T) region. B, Right cassette indicates no presence of CMV IgG (no band at the T region), but that the test is still valid (band at the C region). No serum was added to the IgM strips. Abbreviations: CMV, cytomegalovirus; Ig, immunoglobulin; LFA, lateral flow assay; S, sample port where the sample and buffer are added.

The Healgen CMV Rapid Test Cassette (manufactured by Healgen Scientific LLC, Bellaire, TX, USA) has the capacity to detect both CMV IgM and IgG antibodies in serum, with results read by visual interpretation of colored (pink) band development in the internal strips. The test strip membranes in each cassette are immobilized with CMV antigens on the test region. During the test, the specimen (10 µL of sample, 80 µL of buffer) reacts with colored recombinant mouse antihuman IgG color particle conjugates, which are precoated on the sample pad of the test. The mixture then moves on the membrane by capillary action and interacts with reagents on the membrane. If there are enough CMV antibodies in a specimen, a pink band will form at the test (T) region of the membrane, indicating a positive result. Appearance of a pink band at the control (C) region serves as a procedural control. Results are read at 10 minutes. Figure 2 shows an example image of the Healgen assay. Commercially available test kits (designated “Research Use Only,” not for US market) were purchased for use in this study. This assay is not Food and Drug Administration cleared and is no longer commercially available in the United States.

**Figure 3.**
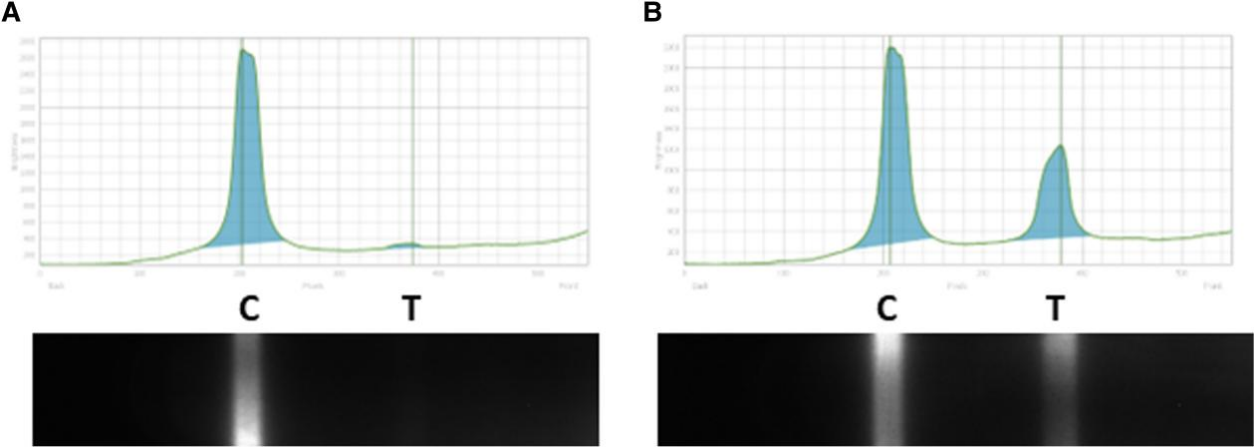
Quulabs’ Qnow results on Qnow Reader. Qoolabs’ QNow CMV Antibody Rapid Test is an immunochromatographic assay that uses antigens to detect CMV antibodies present in a sample. A mixture of antigens representing immunodominant epitopes of CMV and control antibody are immobilized on membrane support as 2 distinct lines. Another mixture of antigens is labeled with europium fluorescent beads to allow visualization of the formation of immunocomplex composed of the CMV antibodies and the antigens. A, Presence of a band at the control region (C) without a band at the test region (T) shows a negative result. B, A band at both the C and T regions indicating a positive result. Abbreviation: CMV, cytomegalovirus.

**Figure 4.**
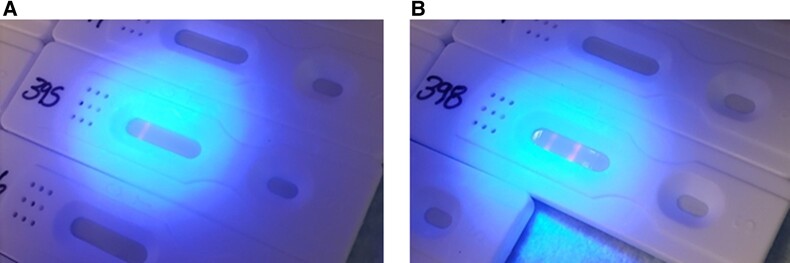
Qoolabs’ Qnow test strips, results read with a UV flashlight. Qoolabs’ QNow assay results using a UV flashlight. Fluorescent bands are visible under UV light (365 nm). A, Presence of 1 band at the C line mark and no band at the T line mark indicates no presence of CMV IgG/IgM, a negative result. B, Presence of 1 band at the C line mark and T line mark indicates the presence of CMV IgG/IgM, a positive result. Results were read at 30 minutes. Safety precautions while using the UV flashlight included avoiding shinning the light directly onto bare skin. Abbreviations: CMV, cytomegalovirus; Ig, immunoglobulin; UV, ultraviolet.

Qoolabs’ QNow CMV Antibody Rapid Test Cassette (manufactured by Qoolabs, Inc., San Diego, CA, USA) is an immunochromatographic assay that uses antigens to detect CMV antibodies present in serum or plasma samples. The test detects IgG from serum, plasma, or whole blood. To perform the test, 10 µL of a sample is applied directly to the sample port of the test cassette, immediately followed by 100 µL of the sample dilution buffer. CMV antibodies present in the sample bind to the antigens labeled with the europium fluorescent beads. The antigen-antibody-bead complexes migrate along the test strip, where they are captured by immobilized detection antigens, forming the test band. Immobilized control antibody captures the overflow complexes, forming the control band. The test results can be read with either the QNow automated reader or under ultraviolet (UV) light (365 nm). Appearance of just the control band indicates a negative result. Appearance of a band at both the control and test regions indicates a positive result. Results are read at 30 minutes. Figure 3 shows an example image from the QNow reader. The original 200 serum samples were read with the QNow automated reader, and a subset of samples (n = 100) were randomly selected from the original serum samples to be interpreted with visual inspection using the QNow UV flashlight. Figure 4 shows an example image using the UV flashlight (365 nm). This assay is commercially available in the United States for “Research Use Only.”

**Figure 5.**
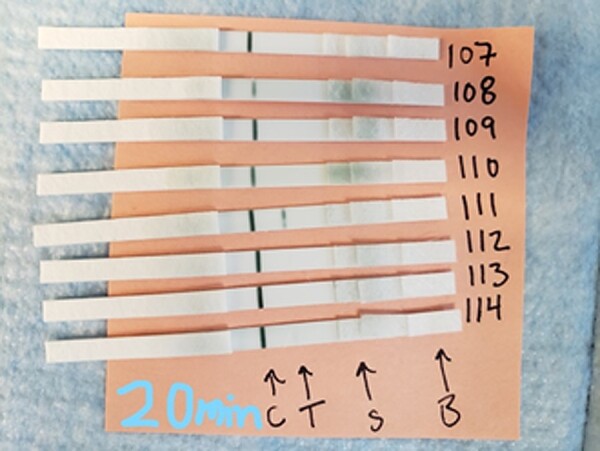
nanoComposix's LFA test strips. nanoComposix assay results. Serum samples are run at a 1:10 dilution with the provided running buffer. The presence of a colored signal at the test line is indicative of CMV IgG species in sample. Sample #111 is positive, while the remaining tests are negative. Results were read at 20 minutes. Abbreviations: B, buffer; C, control; CMV, cytomegalovirus; Ig, immunoglobulin; LFA, lateral flow assay; S, specimen; T, test.

The nanoComposix CMV Antibody Rapid Test Device (manufactured by nanoComposix, Inc., San Diego, CA, USA) is an immunochromatographic assay designed to allow the test line reagents to bind to the target IgGs in the serum sample while the nonspecific IgGs continue up the strip. Serum samples are run at a 1:10 dilution with the provided running buffer. The presence of a colored band at both the control and test lines is indicative of the presence of CMV IgG in a sample. Figure 5 shows an example image of the nanoComposix assay with 8 nanoComposix LFA test strips. Results are visually read at 20 minutes. This assay is not commercially available in the United States.

### Data Analyses

All 3 LFA results were directly compared with the primary reference assay (Zeus ELISA CMV IgG) results. A second reference assay (LIAISON CMV IgG) was performed in the subset of samples that were discrepant between any of the LFAs and the primary reference assay. The overall concordance among the 3 LFAs was evaluated. Sensitivity and specificity were calculated using the following definitions: TP (a true-positive result), TN (true-negative), FN (false-negative), and FP (false-positive). The diagnostic sensitivity {[TP/(TP + FN)] × 100} and specificity {[TN/TN + FP)] × 100} were calculated. Positive predictive value (PPV; the proportion of specimens with positive tests by LFA that were positive by reference test) and negative predictive value (NPV; the proportion of specimens with negative tests by LFA that were negative by reference test) were calculated with the following equations: PPV = {[TP/(TP + FP)] × 100} and NPV = {[TN/(TN + FN)] × 100}. Extrapolated PPV and NPV for 60% CMV seropositivity rate were calculated for each LFA kit.

### Interoperator Variability

**Figure 6.**
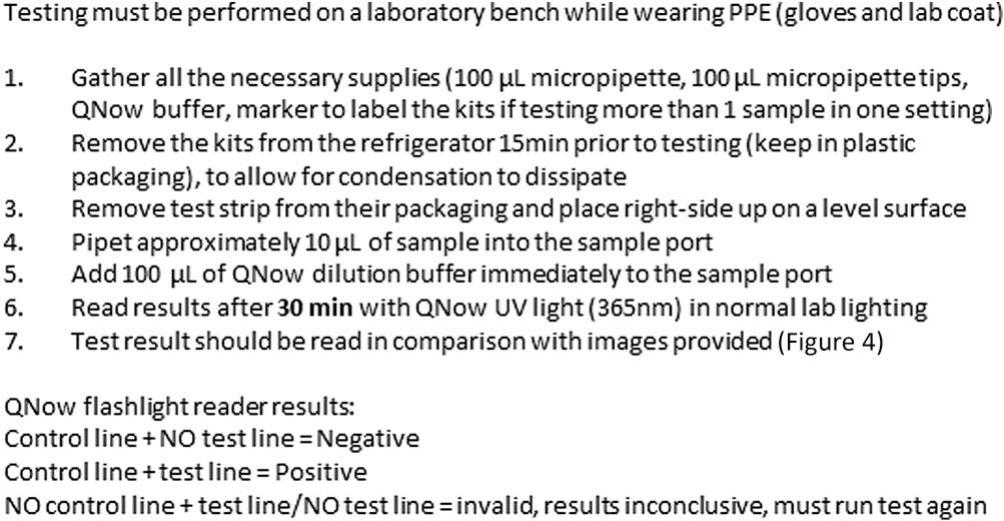
Qoolabs’ QNow CMV IgG LFA SOP. Kits are stored dry at 4°C. Abbreviations: CMV, cytomegalovirus; Ig, immunoglobulin; LFA, lateral flow assay; PPE, personal protective equipment; SOP, standard operating procedure; UV, ultraviolet.

Based on the highest sensitivity and specificity of the QNow assay compared with other tested LFAs, this assay was further assessed for interoperator variability. Ten serum samples (4 negative, 6 positive, result as defined by 100% agreement among the 3 LFAs and the primary reference assay) were analyzed by 5 separate non-laboratory-trained personnel (clinical research coordinators without formal laboratory training). These individuals were blinded to the reference test results. Each operator was provided with detailed step-by-step oral and written instructions including a 5-minute in-person overview of the test procedure, followed by review of the standard operating procedure (SOP) document (Figure 6). The operators independently performed testing without direct supervision while only using the SOP as a guide. They were instructed to take pictures of their results and compare them to the photographic images of positive and negative results provided in the Supplementary Data.

## RESULTS

### Comparison of LFA Test Kits With Reference Assays

Nonduplicate serum samples from 200 consecutive adult patients who had clinically performed CMV testing were included. Ninety-three (46.5%) were seropositive, and 107 (53.5%) were seronegative by the reference Zeus ELISA CMV IgG assay (clinical laboratory result). Table 1 shows a summary of the results of the LFA kits compared with the reference result (Zeus): For 131 (65%) patients/samples, results across all 3 LFAs and the reference assay were concordantly either positive (25%) or negative (40%), and 69 (35%) were discordant in 1 or more of the LFAs. Among these 69 samples for which there was discordance among any of LFAs and the reference assay, 60 (87%) had sufficient volume (>0.5 mL) for testing with a second reference test (LIAISON CMV IgG) (Figure 1). There was 100% agreement between the LIAISON and Zeus CMV IgG reference assays. Summary results for each of the LFAs compared with the Zeus IgG reference assay are shown in Table 2, along with the comparative sensitivity/specificity/PPV/NPV for each of the LFAs (compared with the Zeus IgG reference assay). The sensitivity and specificity and associated 95% CIs for each of the 3 LFAs compared with the ELISA reference assay for serum samples were as follows: 86% (77%–92%)/83% (75%–90%) for Healgen, 99% (94%–100%)/93% (87%–97%) for QNow with the automated reader, and 57% (46%–67%)/97% (92%–99%) for nanoComposix.

**Table 1.** Cumulative CMV IgG Results for Serum Samples (n = 200)

No. of Samples	Summary of Results
50	Positive by Zeus ELISA and all 3 LFA kits
32	Positive by Zeus ELISA and 2 LFA kits
11	Positive by Zeus ELISA and 1 LFA kit
81	Negative by Zeus ELISA and all 3 LFA kits
24	Negative by Zeus ELISA and 2 LFA kits
2	Negative by Zeus ELISA and 1 LFA kit

Sixty of 69 discordant samples had residual volume available for LIAISON CMV IgG testing.

Abbreviations: CMV, cytomegalovirus; Ig, immunoglobulin; LFA, lateral flow assay.

**Table 2.** Comparison of Lateral Flow Assays to the Primary Reference Assay Using Serum (n = 200)

		Reference Assay					
Lateral Flow Assays		Positive	Negative	Sensitivity, %	Specificity, %	PPV, %	NPV, %
Healgen	Positive	80	18	86	83	88	80
	Negative	13	89	95% CI, 77–92	95% CI, 75–90		
QNow	Positive	92	7	99	93	95	98
Automated reader	Negative	1	100	95% CI, 94–100	95% CI, 87–97		
QNow	Positive	36	2	97	97	98	96
UV flashlight^a^	Negative	1	61	95% CI, 85–100	95% CI, 89–100		
nanoComposix	Positive	53	3	57	97	97	60
	Negative	40	104	95% CI, 46–67	95% CI, 92–99		

Abbreviations; NPV, negative predictive value; PPV, positive predictive value.

^a^Only 100 samples were used for the QNow UV flashlight reader.

### Comparison of Zeus ELISA IgG and QNow UV Flashlight

Based on the higher sensitivity/specificity of the QNow assay compared with the other LFAs, and to assess whether visual test interpretation yielded comparable results to those generated by the automated QNow reader, we directly compared the results of the QNow assay interpreted by the automated reader vs visual inspection with a UV flashlight (365 nm) against the reference Zeus assay in a subset of 100 samples. As shown in Table 2, the performance (compared with the reference assay) was similar for the QNow assay interpreted by the automated reader and flashlight methods.

### Serum vs Plasma

Using the Qoolabs’ QNow assay and UV flashlight reader (365 nm), we tested plasma samples (n = 50) to determine whether the sensitivity and specificity would be similar to serum samples. Using the Zeus assay as reference, the sensitivity of the QNow test on plasma samples was 72%, and specificity 96% (Table 3). By comparison, the serum samples’ sensitivity and specificity were 97%/97% (Table 2); results were read under the same UV flashlight and interpreted by the same technician.

**Table 3.** Plasma Results From QNow Assay Read by UV Flashlight Compared With Zeus ELISA Reference Assay (n = 50)

		Zeus ELISA				
		Positive	Negative	Total	Sensitivity, %	Specificity, %
QNow flashlight	Positive	18	1	19	72	96
	Negative	7	24	31	95% CI, 54–90	95% CI, 88–100
	Total	25	25	50		

Abbreviation: UV, ultraviolet.

### Interoperator Variability

We assessed interoperator variability in 10 separate serum samples (4 negative, 6 positive; reference result as defined by concordance among the 3 LFAs and the Zeus IgG reference assay) (Table 4). Among the 50 individual readings, there was agreement for 42 (84%). All operators had concordant results for 5 (50%) of the samples, and among the other 5 samples there was at least 1 discordant reading. Discordant reading by >1 operator occurred for a single sample (ID#5).

**Table 4.** Interoperator Results Compared With Reference Assay (Serum)

Sample ID	Reference Assay Result	Operator 1	Operator 2	Operator 3	Operator 4	Operator 5	% Operators in Agreement With Reference Assay
1	NEG	-	-	-	-	-	100
2	NEG	-	Positive	-	-	-	80
3	NEG	-	-	Positive	-	-	80
4	POS	+	+	+	+	+	100
5	NEG	-	Positive	Positive	-	-	60
6	POS	+	+	+	Negative	+	80
7	POS	+	+	+	+	+	100
8	POS	+	+	+	+	+	100
9	POS	+	+	+	+	+	100
10	POS	Negative	+	+	+	+	80
% individual operators in agreement with reference assay		90	80	80	90	100	

Of the 50 interoperator results, 44 (88%) matched the reference assay results.

## DISCUSSION

We demonstrated significant differences in the performance characteristics of several rapid LFA test kits for determination of CMV serology. We identified Qoolabs’ QNow CMV IgG assay as having good sensitivity, specificity, and consistency of interpretation across operators, making it potentially suitable for rapid on demand single-sample CMV serology testing for planned future clinical trials of CMV reactivation in the ICU setting, or other settings in which rapid turnaround time and single-sample testing might be advantageous. In clinical settings, where the true CMV seropositivity rate is anticipated to be ∼50%–60%, the positive and negative predictive value point estimates of the QNow assay with serum were 93%/99% and 95%/98% with the automated reader and manual visual read with UV flashlight, respectively. However, despite overlapping confidence intervals, the sensitivity of the QNow LFA appeared to be lower in plasma vs serum samples. We also found reasonable agreement for the QNow assay (interpreted by UV flashlight) among operators who did not have prior laboratory training, suggesting that the assay may be suitable for performance by non-laboratory-trained personnel, but this requires further study. Based on the relatively rapid turnaround time and minimal hands-on time and equipment requirements, as well as consistency and ease of interpretation, the QNow lateral flow assay, using the flashlight reader, should be further evaluated as a rapid on demand test for clinical use in settings where a short turnaround time for CMV IgG serology testing may be useful. Specific examples where such assays could potentially be useful include assessment of CMV IgG serology at the time of organ transplant to determine post-transplant CMV risk/preventive strategy, assessment of eligibility (ie, CMV IgG serostatus) for planned future trials for prevention of CMV reactivation in CMV-seropositive ICU patients, or potentially in laboratories with infrequent and/or low-volume testing.

There were several strengths of the study, including a relatively large sample size (n = 200), blinded testing, and the use of established reference standard assays for comparison. Potential limitations include assessment at a single center, use of stored frozen samples, and inclusion of only adult patients. Thus, future studies should include children (in whom both seroprevalence and antibody levels might differ), settings where CMV seropositivity rates might differ from those in the present study, and further assessment of plasma samples. We did not formally assess for interferences in the assays (hemolysis, lipemia, other viral infection, etc.), but the relatively large number of samples tested across a representative population undergoing clinical CMV serology testing, as well as good concordance with a reference assay, provides reassurance that this is unlikely to be a major issue. As the number of operators per sample was small, additional studies to assess interoperator agreement are warranted. Future studies should also assess the reason(s) for the potentially lower sensitivity with plasma vs serum samples with the QNow assay.

In summary, we demonstrated significant variability in the operating characteristics of available CMV IgG LFAs and identified the QooLabs QNow assay as having adequate sensitivity and specificity to justify future studies to define its clinical utility in settings where on demand testing with short turnaround may be useful.
